# *QuickStats:* Percentage* of Adults Aged ≥18 Years Who Have Lost All of Their Natural Teeth,^†^ by Age Group — National Health Interview Survey,^§^ 2000 and 2017

**DOI:** 10.15585/mmwr.mm6822a5

**Published:** 2019-06-07

**Figure:**
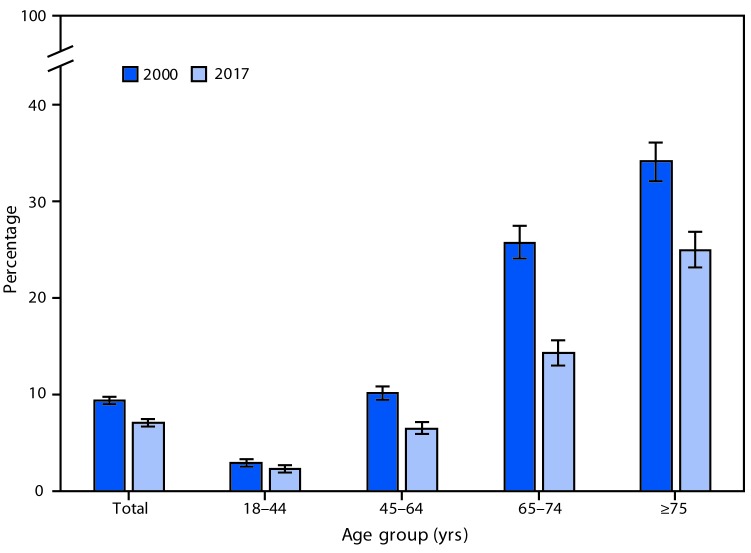
The percentage of adults aged ≥18 years who had lost all of their upper and lower natural teeth decreased from 9.3% in 2000 to 7.0% in 2017, and this pattern was consistent in each age group shown. Complete tooth loss declined from 2.9% to 2.3% among adults aged 18–44 years, from 10.1% to 6.5% among adults aged 45–64 years, from 25.6% to 14.2% among adults aged 65–74 years, and from 34.0% to 24.9% among adults aged ≥75 years.

**Source:** Summary Health Statistics for U.S. Adults: National Health Interview Survey, 2000. https://www.cdc.gov/nchs/data/series/sr_10/sr10_215.pdf. Tables of Summary Health Statistics, 2017. https://ftp.cdc.gov/pub/Health_Statistics/NCHS/NHIS/SHS/2017_SHS_Table_A-6.pdf.

